# The nationwide retrospective cohort study by Health Insurance Review and Assessment Service proves that asthma management decreases the exacerbation risk of asthma

**DOI:** 10.1038/s41598-021-81022-z

**Published:** 2021-01-14

**Authors:** Nam-Eun Kim, Sanghun Lee, Bo Yeon Kim, Ae Gi Hwang, Ji Hyeon Shin, Hyeon-Jong Yang, Sungho Won

**Affiliations:** 1 Department of Public Health Sciences, Graduate School of Public Health, Seoul National University, Seoul, Korea; 2 Department of Medical Consilience, Graduate School of Dankook University, Jukjeon, Korea; 3 Healthcare Review and Assessment Committee, Health Insurance Review and Assessment Service, Wonju, Korea; 4 Chronic Disease Assessment Division, Health Insurance Review and Assessment Service, Wonju, Korea; 5 Quality Assessment Management Division, Health Insurance Review and Assessment Service, Wonju, Korea; 6 SCH Biomedical Informatics Research Unit, Soonchunhyang University Seoul Hospital, Seoul, Korea; 7 Pediatric Allergy and Respiratory Center, Department of Pediatrics, Soonchunhyang University Seoul Hospital, Soonchunhyang University College of Medicine, Seoul, Korea; 8 Interdisciplinary Program of Bioinformatics, Seoul National University, Seoul, Korea; 9 Institute of Health and Environment, Seoul National University, Seoul, Korea

**Keywords:** Asthma, Health policy

## Abstract

Medical costs have recently increased in South Korea due to the rising rate of asthma. Primary clinics serve an important role in asthma management, as they are the first stop for patients presenting with symptoms. The Health Insurance Review and Assessment Service (HIRA) in South Korea has assessed asthma-management quality since 2013, but studies are lacking on whether these assessments have been performed properly and contribute toward reducing asthma exacerbations. Therefore, we investigated whether the HIRA’s quality assessments have decreased asthma exacerbations using national health insurance claims data from 2013 to 2017 of 83,375 primary-clinic and 15,931 tertiary-hospital patients with asthma. These patients were classified into four groups based on disease severity according to the monthly prescribed amount of asthma medication using K-means clustering. The associations between HIRA assessments and asthma exacerbation were analyzed using a generalized estimating equation. Our results showed that exacerbation odds gradually decreased as the HIRA assessments progressed, especially in the mild-severity group, and that exacerbation risk among patients with asthma decreased in the order of assessment grades: “Unsatisfactory,” “Satisfactory,” and “Tertiary.” Therefore, we may conclude that asthma exacerbations may decrease with high quality asthma management; appropriate quality assessment could be helpful in reducing asthma exacerbations.

## Introduction

The global prevalence of asthma has increased by more than 12% since the 1990s, with a much higher prevalence in developed countries and a marked rise in developing countries^1–3^. Especially in regions with a high sociodemographic index quintile, the percentage change in disability-adjusted life years increased by 12.7% during the same period^1,4,5^. Due to economic development in South Korea, the prevalence of asthma has steadily increased by approximately 2.3 times from 1998 to 2013, even after adjusting for age and sex^6^. The socioeconomic burden of asthma, including direct and indirect annual costs, has also increased by approximately USD 15,000 per patient^7–9^. The cost incurred by hospitalization owing to asthma exacerbation can be avoided with high-quality primary care^10^. Therefore, appropriate asthma care practices by primary care providers is important to reduce cases of asthma exacerbation^11–13^. Quality assessments of asthma management have been performed in South Korea since 2013 to improve the quality of asthma management provided by medical institutions. In South Korea, the Health Insurance Review and Assessment Service (HIRA) manages health insurance and medical claims data for 96.6% of the population and facilitates nationwide quality assessments^14^.

The assessment outcome for an institution, as classified by the HIRA, is assigned a “satisfactory” or “unsatisfactory” grade. A total of 11.89% (1066 out of 8967 primary clinics), 14.41% (1278 out of 8866), and 16.19% (1.419 out of 8762) primary clinics were assigned satisfactory grades in the 1st (July 2013–June 2014), 2nd (July 2014–June 2015), and 3rd (July 2015–June 2016) assessment periods, respectively^15^. The outcomes were officially announced each year only for primary clinics, as asthma is an ambulatory care sensitive condition^10^. Primary clinics referred to community hospital with few specialties or just general practice, while tertiary hospitals have highly specialized staff and technical equipment with 300–1500 beds^16^. Asthma management in tertiary hospitals was assumed to be the best among medical institutions, as they had sufficient personnel, facilities, and equipment as prescribed by the ordinance of the Ministry of Health and Welfare. To date, studies on the HIRA assessments have only examined simple descriptive statistics, for example, comparisons of the types of prescribed medications for each stage dependent on the medical institution or comparisons of the pulmonary function test (PFT) ratios^17,18^. In this study, intervention implementation in quality assessment could be tested while collecting evidence of clinical effectiveness that improved asthma management; such a study would be considered a type 3 hybrid design, i.e. a national-scale implementation study without effectiveness studies performed in advance^19^. The satisfactory clinics are assumed to have better prognostics than the unsatisfactory group. As the severe patients might be allocated in satisfactory clinics, there could be more asthma exacerbations in satisfactory clinics. So the quality assessment considered the performance rate of each asthma care indicator rather than exacerbation rate in the classification of satisfactory clinics and it was evaluated whether satisfactory asthma management decreases asthma exacerbation regardless of severity. Therefore, as part of the Joint Project on Quality Assessment Research by HIRA, we aimed to investigate whether asthma management quality assessments by the HIRA effectively classify the institutions and ultimately help encourage proper management to decrease asthma exacerbations.

## Methods

### Study subjects

In this retrospective cohort study, we used the HIRA database to identify whether asthma assessment significantly affected asthma exacerbation. Patients 15 years of age or older diagnosed with asthma (J45 and J46) as a primary or secondary code at least once between July 1, 2013 and June 30, 2017 in the HIRA claims data were eligible for enrolment. Data collection lasted from the first to fourth assessment periods, including data of 4,209,588 patients from the first period (July 2013–June 2014), 4,204,360 from the second (July 2014–June 2015), 4,151,057 from the third (July 2015–June 2016), and 5,288,586 from the fourth (July 2016–June 2017). Therefore, patient data could be repeated in each period. Assessment was performed from the first to the third premeasurement periods, and we evaluated the association between the assessment results and asthma exacerbations 1 year later during the measurement period.

**Figure 1:**
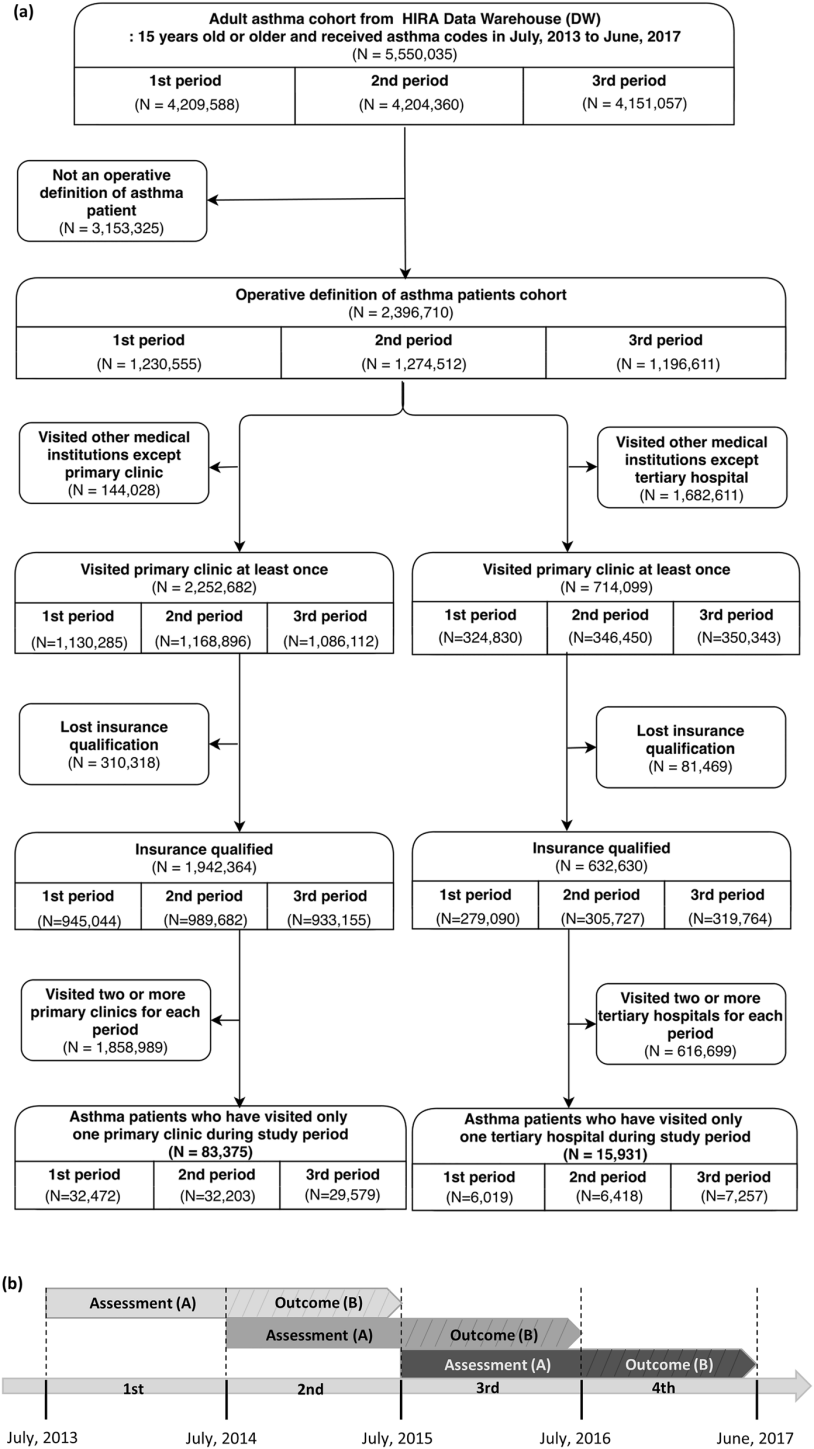
(**a**) Selection process of the study cohort. The study population was categorized as 83,375 primary clinic patients and 15,931 tertiary hospital patients, after all of the following exclusion criteria. (**b**) Retrospective study design. Assessment outcome and other explanatory variables were collected during the premeasurement period (A), and the exacerbation as outcome of assessment was collected the following measurement period (B) for three assessment periods, repeatedly.

In total, 83,375 patients with asthma (32,472, 32,203, and 29,579 patients from the first, second, and third periods, respectively) were selected according to our inclusion and exclusion criteria: (1) more than two outpatient clinic visits while using asthma medication or at least one hospitalization while using oral/intravenous corticosteroids and an outpatient clinic visit while using asthma medication (the list of asthma medications was obtained according to the 2017 Global Initiative for Asthma [GINA] guidelines (Supplementary Table S1)^20^), (2) diagnosed at a primary clinic, (3) had not lost health insurance qualification, and (4) had visited only one primary clinic in any given period. The fourth criterion was adopted because treatment from multiple clinics might overlap, making it difficult to determine the association between HIRA grade and asthma exacerbation. Similarly, 15,931 patients with asthma (6019, 6418, and 7257 patients from the first, second, and third periods, respectively) diagnosed at only one tertiary hospital for each period were selected who also met the first and third criteria (Fig. 1a). There may have been patient overlap; for example, a patient may have visited a primary clinic one year and a tertiary hospital in another year.

### Study design

Assessment grades and asthma severity information from the first to fourth periods were collected during the premeasurement period (A), and asthma exacerbation outcomes were measured during the measurement period (B) of the next year. This was repeated for each period (Fig. 1b); we evaluated whether a hospital with a satisfactory grade in the premeasurement period (A) had less asthma exacerbation cases in the next year’s measurement period (B).

### Outcome and covariate variables

#### Asthma exacerbation

Asthma exacerbation during the measurement period was the primary outcome variable, defined as systemic corticosteroid bursts for asthma treatment based on previous studies^21^ (i.e. more than 80 mg of hydrocortisone or any other corticosteroid of any potency or short-acting beta2 agonists [SABA] nebulizer treatment under J45 and J46 as shown in Supplementary Table S1, obtained from the GINA guidelines). Emergency department visits or hospitalizations for asthma that did not occur in primary clinics were excluded from the definition of asthma exacerbation because HIRA assessment results were only published for primary clinics.

#### Asthma assessment grades

Institutions were classified into three groups: unsatisfactory primary clinics, satisfactory primary clinics, and tertiary hospitals; the quality of asthma management ascended in this order. The assessment outcomes of primary clinics were assigned “satisfactory” or “unsatisfactory” grades and defined according to the following steps. From July to June of every year, patients older than 15 years with asthma (J45 and J46 according to 10th International Statistical Classification of Diseases and Related Health Problems [ICD-10]) were surveyed. The quality assessment was based on seven items: (1) percentage of patients who received the pulmonary function test (PFT) at least once during the assessment period, (2) percentage of ongoing visits (at least three), (3) percentage of patients prescribed inhaled corticosteroids (ICSs), (4) percentage of patients requiring prescribed medicines such as leukotriene receptor antagonists (LTRAs) or ICSs, (5) percentage of patients prescribed long-acting beta2 agonists (LABAs) not using ICSs, (6) percentage of patients prescribed SABAs not using ICSs, (7) percentage of patients prescribed oral corticosteroids snot using ICSs. For the first four items, which were mandatory, a higher score was satisfactory; for the remaining items, a higher score was unsatisfactory. Institutions were assigned a satisfactory grade when they scored higher than the median on each of the mandatory items. However, institutions with scores at the lowest 10% on the remaining three items were excluded. The list of institutions with satisfactory grades is disclosed only in the case of primary clinics, as asthma is a chronic disease for which it is important to prevent acute deterioration and hospitalization through continuous management at a nearby primary medical institution. Although the assessment results of tertiary hospitals were not disclosed, tertiary hospitals are assumed to provide better medical care than primary clinic.

#### Asthma severity

The asthma severity is known as an important confounder of asthma exacerbation and its adjustment is crucial to obtain the unbiased estimates of asthma management effect^22–24^. However, it is difficult to classify asthma severity by diagnosis code, so patients were classified in consideration of their 12-month medication and prescription by using K-means clustering. Each resulting cluster was used as an indicator of asthma severity: From the mildest severity I to the most intense severity IV. First, a rank was assigned according to each principal component code of the GINA guidelines for the drug prescribed throughout the indicated medication period starting on the date of prescription (Supplementary Table S1): Rank 1, low-dose ICS, LTRA, xanthine, or LABA; Rank 2, high-dose ICS, low-dose ICS/LABA; Rank 3, high-dose ICS/LABA; and Rank 4, long-acting muscarinic antagonist inhaler and low-dose oral prednisolone for long-term use^20^. Then, the daily rank-sum was calculated by adding the ranks of all drugs assigned to each day, and the average monthly ranks were determined. Finally, we observed the time-varying pattern of the average monthly rank for each 12-month period. We conducted k-means clustering to distinguish the varying pattern of medication and the degree of severity was classified into four severity clusters for each pattern^25^. The clustering analysis here is described in detail in the Statistical Analyses section. The medications prescribed for asthma exacerbations were not considered in the above severity calculations.

#### Total medication rank

By summing the 365 daily medication ranks, we calculated the yearly total medication rank and consider it as a covariate to control the potential residual effects within the cluster, even after considering the clustered annual asthma severity.

#### Medication possession ratio (MPR)

The MPR reflects the adherence to medication, which tends to reduce the risk of asthma exacerbations^23,26–29^. In administrative claims data, it can be calculated as follows^30–38^.

$${\text{MPR}} = \left( {\frac{{\sum \,the\,number\,of\,days\,treatment\,prescribed\,during\,the\,follow{ - }up\,period}}{follow { - } up\,period}} \right) \times 100$$

The follow-up period denotes the period from the first to the last prescription of asthma medication within the premeasurement period. Observations with an MPR < 20% were categorized as 1 (low adherence 20%–80% as 2, and > 80% as 3 (high adherence. Level 0 indicated the patient had not been prescribed any asthma medication except for alleviating exacerbations during the period.

#### Comorbidity

Comorbidities were adjusted for in the premeasurement period (A) (Fig. 1b) because they may affect asthma exacerbation^39^. Comorbidity variables were defined by whether at least one of the following ICD-10 codes had ever been diagnosed: atopic dermatitis (L20), gastroesophageal reflux disease (K21), chronic rhinitis (J31), allergic rhinitis (J30), chronic sinusitis (J32), depression (F32, F33), anxiety (F40, F41), or obesity (E66)^26^.

### Statistical analyses

Asthma severity was calculated with the average monthly rank based on prescribed drugs for each 12-month period, and the corresponding annual value of severity was obtained. K-means clustering was performed by reflecting these monthly mean values to each dimension. The k-means algorithm was executed using the Euclidean distance, and k was determined to be 4 (the model had the largest overall R-square when k = 4). Chi-square tests were used to examine significant relationships between exacerbations and covariates and *P* values less than 0.05 were considered statistically significant.

#### Model for asthma exacerbation

Associations between risk factors and asthma exacerbations were analyzed with the generalized estimating equation (GEE). PROC GENMOD (SAS version 6.1) and Rex (Version 3.1.3.1) were used to conduct the analysis and generate figures^40^. The logit link function was used because the outcome was binary. There were patients prescribed asthma medications in multiple assessment periods; hence, they may have been repeatedly observed and measured. In such cases, repeated patient measures had a first-order autoregressive (AR[1]) correlation structure according to the period of assessment. We used the AR[1] correlation structure between consecutive measurements on each patient where correlation between two different time points is inversely related with their intervals^41^ and we also found that the results for different correlation structure were similar. Meanwhile, all study subjects had at most one observation per period. With Y_ij_ as 1 if there was exacerbation in the i-th observation of patient j and otherwise 0; we can define $p_{ij} = P\left( {Y_{ij} = 1|X} \right) = E\left( {Y_{ij} } \right)$ with designed matrix X. The model for all patients is as follows.

$$\begin{aligned} logit\left( {p_{ij} } \right) & = \beta_{0} + \beta_{1} Grade_{ij} + \beta_{2} Severity_{ij} + \beta_{3} Totalrank_{ij} + \beta_{4} MPR_{ij} \\ & \quad + \beta_{5} Comorbidity_{ij} + \beta_{6} Period_{ij} + \beta_{7} Sex_{j} + \beta_{8} Age_{ij} + \epsilon_{ij} , \\ \epsilon_{ij} & \sim N\left( {0,\sigma^{2} } \right) \\ \end{aligned}$$

However, the severity group I occupied a very large portion of clusters and they usually visit unsatisfactory clinics more but have less exacerbation than other groups. This can lead to biased estimates of asthma management effects. Hence, we also performed subgroup analyses according to severity for unbiased evaluation of asthma assessment. The final model was selected by comparing the goodness of fit (QICu):

$$\begin{aligned} logit\left( {p_{ijk} } \right) & = \beta_{0} + \beta_{1} Grade_{ijk} + \beta_{2} Totalrank_{ijk} + \beta_{3} MPR_{ijk} + \beta_{4} Comorbidity_{ijk} \\ & \quad + \beta_{5} Period_{ijk} + \beta_{6} Sex_{jk} + \beta_{7} Age_{ijk} + \epsilon_{ijk} , \\ \epsilon_{ijk} & \sim N\left( {0,\sigma_{k}^{2} } \right),\quad k = severity\,cluster\,1, \ldots ,4 \\ \end{aligned}$$

### Ethics statement

The institutional review board of Seoul National University approved this study (IRB No. E1805/003-010). As the data was anonymized, the requirement for written informed consent was waived. All methods were performed in accordance with the relevant guidelines and regulation.

## Results

### Characteristics of all patients

The distribution of each explanatory variable for exacerbations in the following year is shown in Table 1. The number of patients presented in Fig. 1a and the total number of patients presented in Table 1 are inconsistent, as the same patients were repeatedly counted over the periods. There was no significant difference in asthma exacerbation according to assessment period. Male patients had more asthma exacerbations, and age appeared to be positively associated with asthma exacerbations. The proportion of patients with level 0 MPR among those with asthma exacerbations (5.9%) was almost three times that of those without asthma exacerbations (2.1%). Patients with exacerbations had a slightly but significantly lower rate of comorbidities (65.0%) compared to those without exacerbations (67.6%).

**Table 1:** Baseline characteristics of the study population: frequency, proportion, and *P* value from chi-square test for explanatory variables and exacerbation.

Variable	No exacerbation	With exacerbation	Total	*P* value
*Sex*				
Male	46,590 (52.2)*	13,622 (55.2)	60,212	< .0001
Female	42,695 (47.8)	11,041 (44.8)	53,736	
*Age*				
15–34	21,178 (23.7)	3720 (15.1)	24,898	< .0001
35–44	17,436 (19.5)	4326 (17.5)	21,762	
45–54	18,332 (20.5)	5529 (22.4)	23,861	
55–64	14,849 (16.6)	4979 (20.2)	19,828	
65–	17,490 (19.6)	6109 (24.8)	23,599	
*Period*				
1st	30,177 (33.8)	8314 (33.7)	38,491	0.39
2nd	30,329 (34)	8292 (33.6)	38,621	
3rd	28,779 (32.2)	8057 (32.7)	36,836	
*MPR*				
0 (no history)	1833 (2.1)	1446 (5.9)	3279	< .0001
1 (< 20%)	23,061 (25.8)	4901 (19.9)	27,962	
2 (20–80%)	22,612 (25.3)	6886 (27.9)	29,498	
3 (> 80%)	41,779 (46.8)	11,430 (46.3)	53,209	
*Comorbidity*				
Yes	60,332 (67.6)	16,039 (65)	76,371	< .0001
No	28,953 (32.4)	8624 (35)	37,577	
Total	89,285	24,663	113,948	

*Percentage of patients by explanatory variables (%).

### Clustering severity of asthma

**Figure 2:**
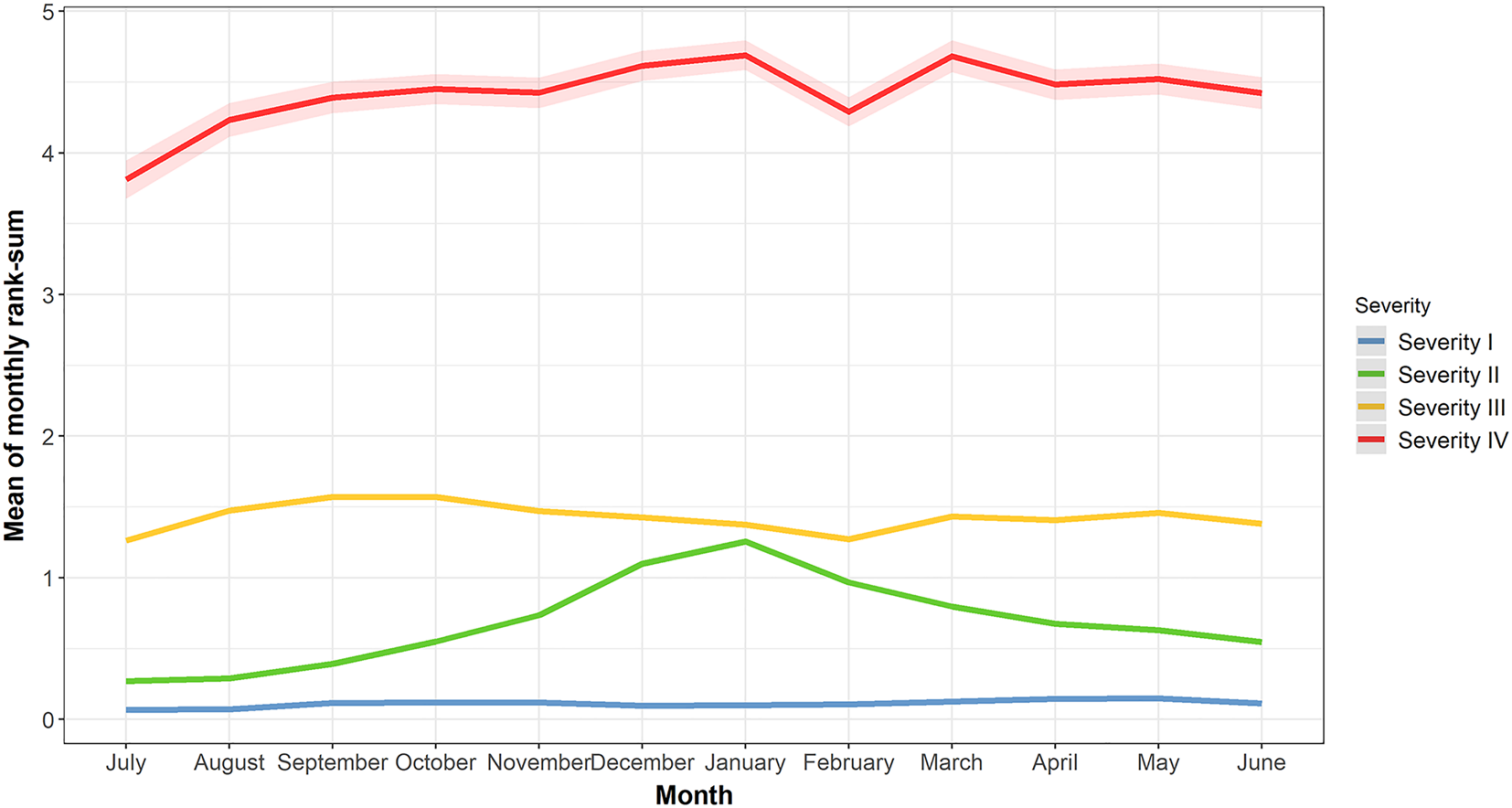
Annual trend of average monthly severity ranks by cluster. The annual severity trends by k-means clustering of 12-month average rank-sum and their 95% confidence intervals.

As a result of the clustering analysis, the patients with asthma were classified into four groups according to their overall severity. Severity group I (n = 83,175) showed the lowest severity, with a stable pattern, and included most patients (Fig. 2). The 83,175 patients in severity group I comprised 35.1% of the patients from the first assessment period, 34.1% from the second, and 30.8% from the third. Severity group II (n = 11,116) showed a rapidly increasing pattern in winter, peaking in January and gradually decreasing as the weather improved. However, it still showed the second lowest overall severity among the four groups. Severity group III (n = 16,692) showed moderate and stable severity throughout the year. Severity group IV comprised only 2965 patients, but they had high severity throughout the year. They also showed a severity peak in January and relatively low severity during the summer season. Notably, the number of patients included in severity group IV increased over the periods, as opposed to the distribution in severity group I (Table 2). The distribution of exacerbations in the following year according to severity clustering showed that the exacerbation rate gradually increased as severity increased (Table 3, Supplementary Fig. S1).

**Table 2:** Severity distribution of total study population according to the assessment period.

Period	Severity I	Severity II	Severity III	Severity IV	Total
1st	29,179 (35.1)*	3681 (33.1)	4812 (28.8)	819 (27.6)	38,491
2nd	28,390 (34.1)	3503 (31.5)	5721 (34.3)	1007 (34.0)	38,621
3rd	25,606 (30.8)	3932 (35.4)	6159 (36.9)	1139 (38.4)	36,836
Total	83,175 (73.0)	11,116 (9.8)	16,692 (14.6)	2965 (2.6)	113,948

*Percentage of patients by assessment period (%).

**Table 3:** Exacerbation in the following year according to severity of total study population.

Severity	No exacerbation	With exacerbation	Total	*P* value
I	69,424 (83.5)*	13,751 (16.5)	83,175	
II	7993 (71.9)	3123 (28.1)	11,116	< .0001
III	10,441 (62.6)	6251 (37.4)	16,692	
IV	1427 (48.1)	1538 (51.9)	2965	
Total	89,285	24,663	113,948	

*Percentage of patients by exacerbation (%).

**Figure 3:**
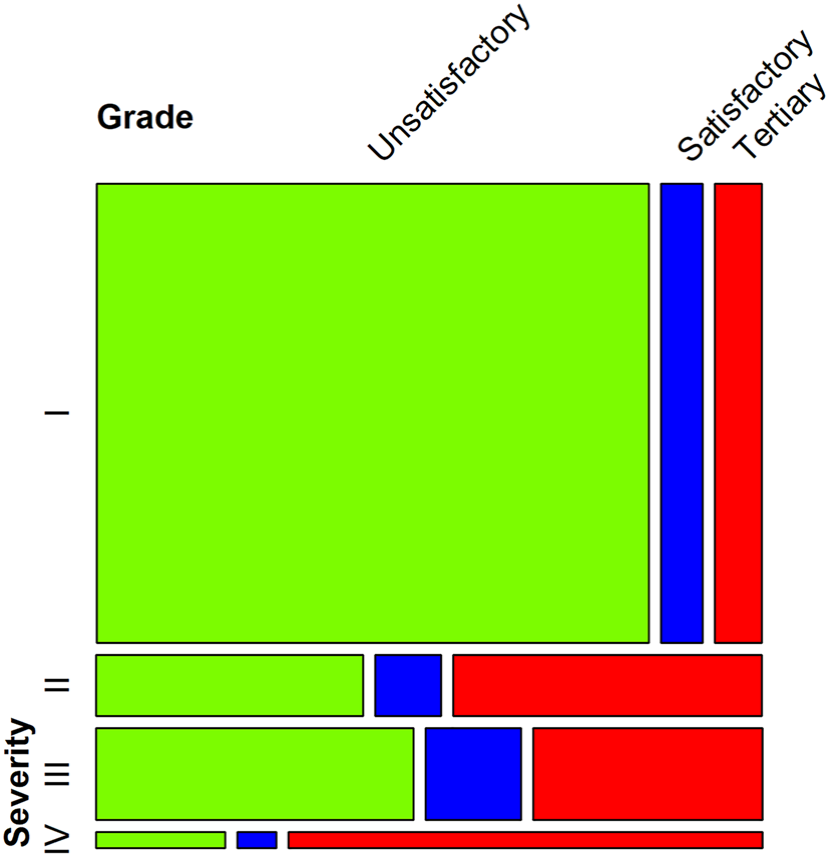
Severity distribution according to the assessment grade of visited medical institutions. Mosaic plot showing the distribution of assessment grade and severity.

In addition, the distribution of assessment grades according to asthma severity, presented in Fig. 3, showed that severity group I accounted for the largest portion of patients. As a result, the overall analysis result can be determined according to the result of severity group I. Also, the higher the severity, the higher the rate of visits to a hospital with better grade. This selection bias can lead to more exacerbations in better hospitals. Therefore, subgroup analyses were conducted to correct for this bias.

### GEE model selection and effect of assessment

The results of the GEE analyses of risk factors associated with asthma exacerbation are shown in Tables 4 and 5, wherein estimated values of the odds ratios (ORs) and *P *values are described. The statistics for the periods and grades were presented according to severity subgroup in the subgroup analyses. Total QICu can be calculated by summing the QICus at each severity level. In the whole-group model, a higher OR was observed in the satisfactory-grade clinics than in those with unsatisfactory grades (Table 4). The OR of the grade was different from before considering severity as a covariate (results are not attached). The estimates of covariates other than asthma management can be biased unless their confounders were considered properly. However, the direct effect of assessment period showed the OR decreased over time significantly, with *P *values lower than 0.0001 over time. The OR of exacerbation significantly increased with the severity of asthma, except for the highest severity. Further, men showed a higher degree of asthma exacerbation than women, but the difference was not significant (*P* = 0.09). Patients younger than 45 years had a significantly lower degree of exacerbation than patients older than 65 years (*P* < 0.0001). Regarding MPR, exacerbations were more frequent in the group with adherence of 80% or lower than in the group with adherence of > 80% (all *P* < 0.0001). In particular, the group with 0 MPR, with conditions entirely unmanaged by asthma medication, had a large OR of exacerbation as a result of the lack of proper management. Patients without comorbidities were found to have a lower OR of exacerbation than those with comorbidities (*P* = 0.04) (Supplementary Table S2).

**Table 4:** OR and 95% confidence interval (CI) of grades to exacerbation in whole-group model (QICu = 110,830).

Variable	OR	95% lower CI	95% upper CI	*P* value
*Grade*				
Unsatisfactory	1.08	1.02	1.13	0.01
Satisfactory	1.23	1.15	1.32	< .0001
Tertiary	Reference

**Table 5:** OR and 95% confidence interval (CI) of covariates to exacerbation in subgroup model (QICu = 109,430).

Severity	Variable*	OR	95% lower CI	95% upper CI	*P* value	QICu
I	*Period*					70,652
	1st	1.41	1.34	1.49	< .0001	
	2nd	1.13	1.06	1.19	< .0001	
	*Grade*					
	Unsatisfactory	1.1	1.02	1.2	0.02	
	Satisfactory	1.22	1.1	1.36	0.0002	
II	*Period*					12,987
	1st	1.14	1.03	1.26	0.01	
	2nd	1.05	0.95	1.17	0.36	
	*Grade*					
	Unsatisfactory	1.37	1.22	1.53	< .0001	
	Satisfactory	1.26	1.08	1.47	0.004	
III	*Period*					21,733
	1st	1.03	0.96	1.1	0.47	
	2nd	0.98	0.92	1.05	0.6	
	*Grade*					
	Unsatisfactory	1.35	1.24	1.47	< .0001	
	Satisfactory	1.21	1.08	1.35	0.0006	
IV	*Period*					4058
	1st	1.04	0.87	1.25	0.66	
	2nd	0.92	0.77	1.11	0.38	
	*Grade*					
	Unsatisfactory	1.12	0.91	1.37	0.29	
	Satisfactory	1.24	0.9	1.71	0.2	

*Reference levels: 3rd for Period variable, tertiary for Grade variable.

In the final model, controlling for the bias due to large cluster I, the QICu value (109,430) signified a better model with a lower value than that of the whole-group model (Table 5). The cluster-wise analysis of the entire dataset showed statistically significant outcomes of the grade variable with ORs larger than 1 in severity clusters I, II, and III. The OR of clinics with unsatisfactory grades was larger than that of clinics with satisfactory grades in the case of clusters II and III, suggesting that poor asthma management was positively associated with asthma exacerbations. In cluster IV, the ORs were larger than 1 but statistically insignificant. In addition, the OR of exacerbation in terms of period significantly decreased over time in cluster I, which also suggests exacerbation was reduced in the mildest severity group, as the assessment was repeated every year.

## Discussion

Our large cohort study showed that institution assessment grade, severity, sex, age, MPR, comorbidities, and assessment itself may be important factors related to asthma exacerbations. Moreover, the magnitude of the effect of grade on asthma exacerbation varied in the subgroup analysis according to severity. We compared primary outcomes of asthma patients between satisfactory primary clinics and tertiary hospitals to accurately determine the effect of severity on the exacerbation, with the trend of severe patients being concentrated in satisfactory clinics more than in unsatisfactory clinics.

All patients could be clustered into four severity groups. In severity groups I-IV, 6185 (7.4%), 5357 (48.2%), 5963 (35.7%), and 2189 (73.8%) patients visited tertiary institutions, respectively (Table 3, Table S4). As shown in Fig. 2, severity groups II and IV showed a common peak in January, with severity group IV decreasing in February and peaking again in March. Therefore, seasonality was observed in our data, suggesting that seasonality or air pollution, risk factors for asthma exacerbation, may affect the severity of asthma^42,43^. Based on the results of a long-term monthly time-series analysis from 2000 to 2014, fine dust concentration (PM10) is highest in March in South Korea^44^. Therefore, the second peak in March for the highest asthma severity group could be explained by the effect of air pollution. Meanwhile, we included years as a covariate to adjust the national trends and the number of patients with asthma exacerbation tends to slightly decrease.

The asthma exacerbation rate decreased as the HIRA assessment progressed in this study, although this was only observed in severity group I in the final subgroup model. As each primary clinics’ grade is disclosed in public, asthma patients can select and visit the satisfactory clinic nearby and this may have made hospitals strive to provide higher health care and receive satisfactory grades. Actually, the percentage of clinics that were all in the top 50% of the previous 4 items and not in the bottom 10% of the last 3 items and met the satisfactory grade increased from 11.89% to 16.79% as assessment period passed^15^. However, financial rewards and penalties based on assessment results as applied to the results of the quality assessment of other diseases (high blood pressure, diabetes, hemodialysis) are not yet utilized for patients with asthma. The HIRA assessment itself might be more helpful in improving the quality of asthma management to prevent asthma exacerbation and alleviate asthma severity if administrators applied a similar incentive system for patients with asthma.

According to HIRA reports, each of the seven indicators had its own basis for selection and improved as the assessment experience increased. Adhering to these guidelines is known to reduce the risk of asthma exacerbation, and hospitals that comply with the 7 items are expected to better prevent exacerbations^26,45,46^. Due to the imbalance of asthma severity between grades, the quality assessment program did the best to encourage improving performance rate, did not consider the exacerbation rate in the classification of satisfactory clinics. HIRA does not disclose the individual performance rates each medical institution had for each item, but it presents three-year statistics as the HIRA report^15^. It also provides rationale for selection as the evaluation criteria for each item as follows. (1) The total percentage of patients who received the PFT at least once during the assessment period was included because, when asthma is diagnosed, the most useful indicator of future risk is pulmonary function, which requires periodic tests of lung function not only at the time of diagnosis, but also 3–6 months after treatment and during follow-up. The PFT rate of the third period of assessment increased 4.87% compared to that of the first period. Tertiary hospitals showed 85.44%, while primary clinics showed 20.09%^15,45^. (2) The percentage of ongoing visits (at least three) was considered as an indicator of a favorable hospital; regular visits from patients allow for regular monitoring of their asthma^46^. (3) and (4) The percentage of patients requiring prescribed medicines such as LTRAs or ICSs was included, because inhaled steroids are the most effective prophylactic drug for maintaining asthma control and should be used if possible for all patients with asthma. The use of anti-leukotriene alone is less effective than low-dose inhaled steroids. However, in some cases, it can be used as initial maintenance treatment. The proportion of patients prescribed ICS or LTRA in the third period was 63.65%, increased 4.50% from the first period^47,48^. (5), (6) and (7) The percentage of patients prescribed LABAs/SABAs or oral corticosteroids not using ICSs should be low to get a satisfactory grade. This is because beta2 agonists or oral corticosteroids need be used with inhaled steroids, used in urgent situations such as acute exacerbations, or used with various restrictions and caution of abuse^49,50^. These last three items showed decreasing trends over the periods according to HIRA report^15,18^.

For severity groups II and III, the patients managed in primary clinics had more exacerbations than had those managed in tertiary hospitals; the patients managed in unsatisfactory-grade primary clinic had the most exacerbations. In contrast, severity groups I and IV required in-depth analyses. Severity group I was composed of patients with mild asthma, ranking close to 0 throughout the year. As such, they did not require medicated most of the time, but experienced exacerbation sometimes. This made it hard to classify severity among patients with mild asthma and control the residual effects of each patient’s severity in the model. Although the OR of the satisfactory group was larger than that of the unsatisfactory group, their confidence intervals overlapped. We can conclude there was no significant difference between satisfactory and unsatisfactory clinics among severity group I in terms of asthma exacerbation. For severity group IV, it was also difficult to judge whether (1) the characteristics of the patients differed, (2) the results were not significant because the sample was too small, or (3) the current classification criteria were insufficient for severity group IV. There were only 776 patients in severity group IV who visited primary clinics, and 435 of those had exacerbations (Supplementary Table S4).

Our study had several strengths. There were previously only reviews of reports published by the HIRA regarding qualitative assessments^18,51,52^, but we actively evaluated the effectiveness of the asthma management and the policy through quantitative analysis using raw data from health insurance claims. Also, this quantitative statistical analysis applied a clustering algorithm to analyze the asthma severity pattern in large cohort data. Asthma severity, one of the important confounding variable of asthma exacerbation, was calculated by rank according to the GINA guideline, and patients with asthma were grouped according to the rank-sum values, unlike in previous studies that only utilized clinical findings and hospital data with small numbers of patients^14,24,53^. The individual effect was also considered in the statistical model with a correlation structure. As such, the characteristics of each patient could be more accurately considered using the 3-year data with repetition.

However, our study also had several limitations. There is a possibility of incomplete coding accuracy and recording, as the measurement of asthma medication and diagnosis of exacerbation was based on claims data. A previous study on the validity of health claims data in Korea using ICD-10 codes showed primary and secondary codes on 9278 claims had accordance rates of only 82.0% and 56.4%, respectively^54^. Secondly, because this was an observational study, all of the confounding factors could not be included in the model, leading to residual confounding. Many variables affecting the results, including smoking status, medical records, and socioeconomic status, were not fully available in the HIRA database. Despite the seasonality of asthma severity due to air pollution in our data (Fig. 2), patient residence was not included in the HIRA database due to privacy issues. Thus, the air pollution factor was not included in the model. Finally, asthma exacerbation was defined on the basis of prescription medications, but emergency room visits or hospitalizations due to asthma should be included^21,26,28^. However, admission and emergency room visits could be excluded from the definition, as the major concern of this study was the quality assessment grades of primary clinics.

In conclusion, asthma exacerbation is affected by the quality of asthma management and its assessment, and our findings suggest that proper asthma management can reduce asthma exacerbations, regardless of asthma severity (Table 5). Thus, evidence has been provided for the clinical effectiveness of and justification for quality assessment based on national-scale policy implementation^19^. However, as the effectiveness of asthma management could not be analyzed properly for patients with the lowest severity, new criteria should be established in further study to clearly subdivide asthma severity^11–13^.

## Supplementary Information

Supplementary Information.

## Data Availability

The data that support the findings of this study are available from the HIRA. Restrictions apply to the availability of these data, which were used under license for the current study, and so are not publicly available due to personal information protection. Data are available at https://opendata.hira.or.kr/ with the permission of the HIRA.
