# Supplementary material for: The nationwide retrospective cohort study by Health Insurance Review and Assessment Service proves that asthma management decreases the exacerbation risk of asthma
Source: Sci Rep. 2021 Jan 14;11:1442. doi: 10.1038/s41598-021-81022-z (PMC7809363; doi:10.1038/s41598-021-81022-z)
Supplement: Supplementary file 1 — Supplementary Information. [file 41598_2021_81022_MOESM1_ESM.pdf]

# The nationwide retrospective cohort study by Health Insurance Review and Assessment Service proves that asthma management decreases the exacerbation risk of asthma.

*Nam-Eun Kim, Sanghun Lee, Bo Yeon Kim, Ae Gi Hwang, Ji Hyeon Shin, Hyeon-Jong Yang, and Sungho Won*

## SUPPLEMENTARY FIGURE

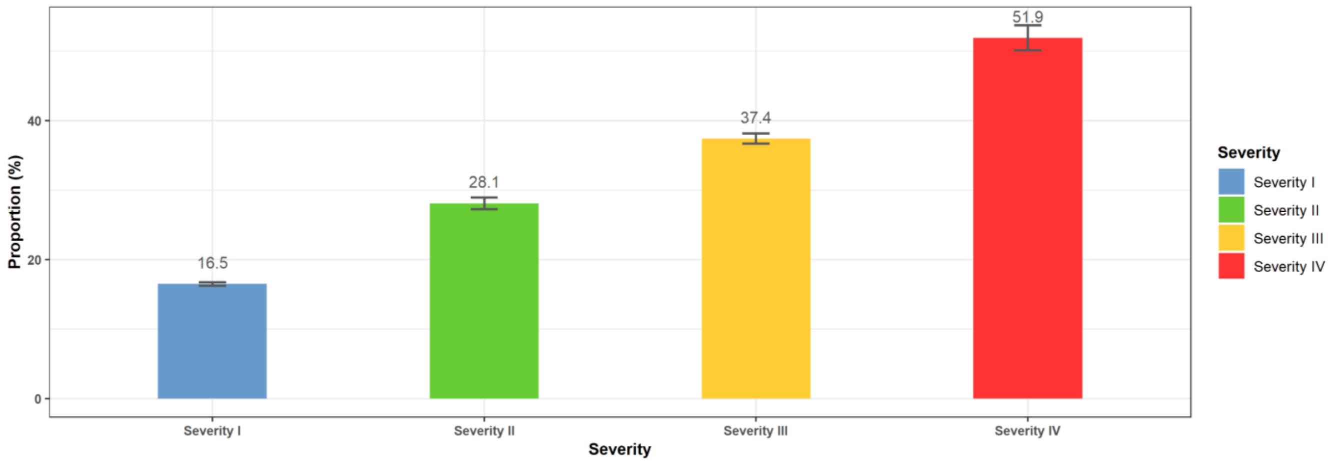


**Supplementary Figure S1.** Exacerbation rate in each severity cluster**.** Exacerbation rate and its 95% confidence interval in each severity cluster.

**SUPPLEMENTARY TABLES**

***Supplementary Table S1***. *The list and rank of asthma medication according to the GINA guideline*

| Component | Type | Rank | Prescription period | Exacerbation |
|---|---|---|---|---|
| **LTRA** | | | | |
| Montelukast | Oral | 1 | 1 | 0 |
| Pranlukast | Oral | 1 | 1 | 0 |
| Zafirlukast | Oral | 1 | 1 | 0 |
| **Xanthine** | | | | |
| Aminophylline | Oral | 1 | 1 | 0 |
| Aminophylline | intravenous | 1 | 1 | 0 |
| Theophylline | Oral | 1 | 1 | 0 |
| Bamiphylline | Oral | 1 | 1 | 0 |
| Diethylaminoethyltheophylline | Oral | 1 | 1 | 0 |
| Oxtriphylline | Oral | 1 | 1 | 0 |
| doxofylline | Oral | 1 | 1 | 0 |
| **LABA** | | | | |
| Bambuterol | Oral | 1 | 1 | 0 |
| Clenbuterol | Oral | 1 | 1 | 0 |
| Formoterol | Oral | 1 | 1 | 0 |
| Tulobuterol | patch | 1 | 1 | 0 |
| **ICS** | | | | |
| Low-dose ICS | | | | |

| | | | | |
|---|---|---|---|---|
| Budesonide | Inhaler | 1 | 30 | 0 |
| Ciclesonide | Inhaler | 1 | 30 | 0 |
| beclomethasone | Inhaler | 1 | 1 | 0 |
| Fluticasone propionate | Inhaler | 1 | 30 | 0 |
| Medium- to high-dose ICS | | | | |
| Budesonide | Inhaler | 2 | 30 | 0 |
| Fluticasone propionate | Inhaler | 2 | 30 | 0 |
| **ICS/LABA** | | | | |
| Formoterol | Inhaler | 2 | 30 | 0 |
| Fluticasone & Vilanterol | inhaler | 2 | 30 | 0 |
| **Systemic corticosteroid** | | | | |
| Betamethasone < 2.4mg | | 4 | 1 | 1 |
| Deflazacort < 30mg | | 4 | 1 | 1 |
| Dexamethasone < 3mg | | 4 | 1 | 1 |
| Hydrocortisone < 80mg | | 4 | 1 | 1 |
| Methylprednisolone < 16mg | | 4 | 1 | 1 |
| Prednisolone < 20mg | | 4 | 1 | 1 |

***Supplementary Table S2.*** *OR and 95% confidence interval (CI) of adjusting covariates to exacerbation in whole-group model*

| **Variable** | **OR** | **95% Lower CI** | **95% Upper CI** | **P-value** |
|---|---|---|---|---|
| **Period** | | | | |
| 1st | 1.28 | 1.23 | 1.33 | <.0001 |
| 2nd | 1.09 | 1.05 | 1.13 | <.0001 |
| 3rd | reference | | | |
| **Severity** | | | | |
| II | 1.7 | 1.6 | 1.81 | <.0001 |
| III | 1.87 | 1.73 | 2.02 | <.0001 |
| IV | 1.01 | 0.83 | 1.22 | 0.9401 |
| I | reference | | | |
| **Total rank** | 1.0013 | 1.0011 | 1.0014 | <.0001 |
| **Sex** | | | | |
| Male | 1.03 | 1 | 1.06 | 0.09 |
| Female | reference | | | |
| **Age** | | | | |
| 15- 34 | 0.73 | 0.69 | 0.77 | <.0001 |
| 35-44 | 0.93 | 0.88 | 0.97 | 0.003 |
| 45-54 | 1.03 | 0.98 | 1.08 | 0.24 |
| 55-64 | 1.07 | 1.02 | 1.12 | 0.007 |
| 65- | reference | | | |
| **MPR** | | | | |
| 0 (No history) | 4.75 | 4.35 | 5.2 | <.0001 |
| 1 (-20%) | 1.62 | 1.55 | 1.7 | <.0001 |
| 2 (20%-80%) | 1.69 | 1.62 | 1.76 | <.0001 |
| 3 (80%-) | reference | | | |
| **Comorbidity** | | | | |
| No | 0.96 | 0.93 | 1 | 0.04 |
| Yes | reference | | | |

***Supplementary Table S3.*** *Exacerbation in the following year according to severity of the tertiary hospital patients*

| **Severity** | **No exacerbation** | **With exacerbation** | **Total** | **P-value** |
|---|---|---|---|---|
| I | 5,041 (81.5) * | 1,144 (18.5) | 6,185 | <.0001 |
| II | 4,060 (75.8) | 1,297 (24.2) | 5,357 | |
| III | 3,875 (65) | 2,088 (35) | 5,963 | |
| IV | 1,086 (49.6) | 1,103 (50.4) | 2,189 | |
| **Total** | 14,062 | 5,632 | 19,694 | |

*(): percentage of patients by exacerbation (%)

***Supplementary Table S4.*** *Exacerbation in the following year according to severity of primary clinic patients*

| **Severity** | **No exacerbation** | **With exacerbation** | **Total** | **P-value** |
|---|---|---|---|---|
| I | 64,383 (83.6) * | 12,607 (16.4) | 76,990 | <.0001 |
| II | 3,933 (68.3) | 1,826 (31.7) | 5,759 | |
| III | 6,566 (61.2) | 4,163 (38.8) | 10,729 | |
| IV | 341 (44) | 435 (56) | 776 | |
| **Total** | 75,223 | 19,031 | 94,254 | |

*(): percentage of patients by exacerbation (%)
